# PENELOPE-CTRL: protocolised LDL-C lowering compared to real-world care in patients after myocardial infarction

**DOI:** 10.1007/s12471-025-01964-1

**Published:** 2025-07-01

**Authors:** Tinka van Trier, Aaram Omar Khader, Sander van der Brug, Anho Liem, Astrid Schut, Jan Tijssen, Fabrice Martens, Marco Alings, the PENELOPE study group

**Affiliations:** 1 Department of Cardiology, Amsterdam University Medical Centers, Amsterdam, The Netherlands; 2 Department of Cardiology, Erasmus Medical Centre, Rotterdam, The Netherlands; 3 Department of Cardiology, Amphia Hospital, Breda, The Netherlands; 4 Department of Cardiology, Franciscus Gasthuis, Rotterdam, The Netherlands; 5 WCN (Dutch Network for Cardiovascular research), Utrecht, The Netherlands; 6 Department of Cardiology, Deventer Hospital, Deventer, The Netherlands

## What’s new

- Real-world LDL-cholesterol care in the Netherlands falls short, as evidenced by infrequent LDL‑C measurements, low-intensity treatment combinations, and unmet targets.
- A short protocol-led strategy to LDL-cholesterol lowering in patients 18–70 years after myocardial infarction was found to be superior compared to real-world practice, resulting in respectively 86% vs. 55% patients reaching targets of ≤ 1.8 mmol/L during the protocol period of 6 months (*p* < 0.001)
- Despite a reduction in this effect over time, with 64% vs. 55% maintaining target after one year follow-up, the benefits remained statistically significant after one year (*p* < 0.001), highlighting the added value of protocol-led LDL‑C management in patients after myocardial infarction.

## Introduction

Effective lowering of low-density lipoprotein cholesterol (LDL-C) is a cornerstone in reducing cardiovascular risk in patients with atherosclerotic cardiovascular disease (ASCVD) [1]. Despite evidence-based guidelines recommending intensive LDL‑C management, achieving target LDL‑C levels in clinical practice remains a challenge [2–5].

The PENELOPE study (2019–2020) demonstrated that a protocol-led LDL‑C lowering strategy in post-myocardial infarction (MI) patients enabled nearly 90% to reach the Dutch LDL‑C target of ≤ 1.8 mmol/l [6], with almost two-thirds maintaining this target after one year [7]. The current study (PENELOPE-CTRL) compares these results with real-world LDL‑C target attainment in post-MI patients in the Netherlands.

## Methods

### Study design and population

PENELOPE was a prospective cohort study conducted between 2019 and 2020, including patients with a recent myocardial infarction (MI) and documented ASCVD and/or diabetes across 23 non-academic centres in the Netherlands. A predefined, stepwise LDL‑C lowering protocol was implemented: initially, high-intensive statin (HIST) therapy was initiated (step 1); if necessary, ezetimibe was added (step 2); and if LDL‑C levels remained > 1.8 mmol/L after ≥ 4 weeks, treatment was escalated to a PCSK9i inhibitor (step 3) [6].

The comparator group, CTRL, included retrospectively selected patients from 13 non-academic centres during the same period (Electronic Supplementary Material, Fig. S1). Apart from an age cap of 70 years, applied to minimise the inclusion of frail individuals, inclusion criteria were identical to those in PENELOPE, i.e. admission for type I (N)STEMI and a history of diabetes mellitus and/or ASCVD. In CTRL, continuous LDL‑C was collected from 3 months prior to index MI and 18 months post-MI.

### Outcome definitions

The primary outcome was the proportion of patients achieving LDL-C ≤ 1.8 mmol/L within 6 months post-MI (‘protocol period’), compared between the PENELOPE ≤ 70 and CTRL cohorts. Secondary outcomes included LDL‑C target attainment at one year post-MI and any point during follow-up. Additional comparisons were made between median LDL‑C levels, time to target attainment of the stricter European LDL‑C target (≤ 1.4 mmol/L).

### Statistical methods

Potential baseline imbalances were assessed. Proportions of patients achieving LDL‑C targets were compared using Fisher exact test, with 95% confidence intervals calculated using Wilson’s score interval (detailed methods in Electronic Supplementary Material, Box S2).

## Results

Of the 827 CTRL patients, 24% were women, 36% had a STEMI and 73% had a history of ASCVD. Baseline characteristics showed no clinically relevant differences compared with the PENELOPE ≤ 70 cohort (Electronic Supplementary Material, Tab. S1), with similar baseline use of lipid-lowering therapy (Electronic Supplementary Material, Tab. S2) and approximately one-third of patients already at target (32% in PENELOPE ≤ 70 vs. 33% in CTRL, *p* = 0.7). LDL‑C measurements were available for 663 (100%) of PENELOPE ≤ 70 participants versus 759 (92%) of CTRL patients.

**Fig. 1:**
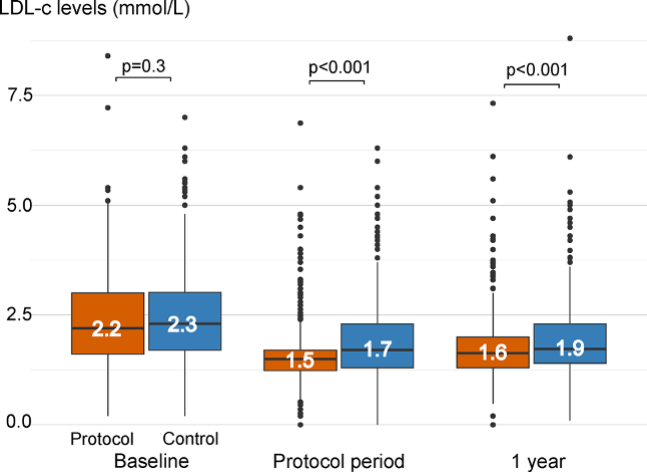
Distribution and median of LDL‑C levels with a short protocol-led LDL‑C lowering strategy (PENELOPE ≤ 70) versus real-world controls (CTRL) in patients after myocardial infarction. At baseline (hospitalisation for myocardial infarction), within the study period of 6 months and after approximately one year

Main comparisons of LDL-C levels are visualised in Figure 1. Within 6 months, 86% of PENELOPE ≤ 70 patients achieved LDL-C ≤ 1.8 mmol/L vs. 55% in CTRL (*p* < 0.001). The median (IQR) LDL‑C was 1.5 mmol/L (1.2–1.7) in PENELOPE ≤ 70 vs. 1.7 mmol/L (1.3–2.3) in CTRL, *p* < 0.001.

At one year, target attainment remained higher in PENELOPE ≤ 70 compared to CTRL (64% vs. 55%, *p* = 0.008), with lower median (IQR) LDL‑C levels in PENELOPE ≤ 70 (1.6 [1.3–2.0]) compared to CTRL (1.9 [1.4–2.3]), *p* < 0.001; (Electronic Supplementary Material, Tab. S3).

Through complete follow-up, 88% (95% CI: 86–91%) of PENELOPE ≤ 70 vs. 65% (95% CI: 62–69%) in CTRL achieved target LDL‑C (*p* < 0.001). The median time to target for those not at target at baseline was 43 days (3–77) in PENELOPE ≤ 70 vs. 51 (2–176) days in CTRL (*p* < 0.001). The stricter LDL‑C targets (≤ 1.4 mmol/L) were achieved in 43% (95% CI: 40–47%) of PENELOPE ≤ 70 patients vs. 41% (95% CI: 37–45%) of CTRL (*p* = 0.4).

## Discussion

The PENELOPE-CTRL study showed that a protocol-led LDL‑C lowering strategy significantly improved LDL‑C goal attainment compared to real-world controls in the Netherlands. High LDL‑C target attainment with the protocol (86% initially and 64% at one year post-MI) contrasted with only 55% in routine care.

Poor LDL‑C goal attainment and infrequent LDL‑C measurements in CTRL aligns with findings from recent European studies (DA VINCI, EUROASPIRE V, SANTORINI, INTERASPIRE), highlighting a critical gap in LDL‑C monitoring in current real-world practice. A sustained LDL‑C reduction of 0.3 mmol/L after one year, relative to real-world controls, translates into an estimated 3% additional annual reduction in cardiovascular event risk [1, 8]. Continued adherence to LDL‑C lowering may amplify risk reduction over time.

Strengths of this study include the contemporary, geographically representative nationwide cohorts. However, the retrospective design of PENELOPE-CTRL may introduce bias, for example, due to the observed lack of LDL‑C monitoring in routine care. Sensitivity analysis using best- and worst-case imputation for missing LDL‑C data showed superiority of PENELOPE ≤ 70 over CTRL in both scenarios (*p* < 0.001).

In conclusion, the PENELOPE-CTRL study illustrates that a protocol-led LDL‑C lowering strategy significantly improves both timely and overall LDL‑C goal attainment compared to real-world controls. A graphical summary (in Dutch) of the study’s key methods, results, and implications has been developed for use in clinical settings to raise awareness and support the implementation of structured LDL‑C management in patients after myocardial infarction to optimise clinical outcomes (Electronic Supplementary Material, Fig. S4).

## Supplementary Information

Boxes S1 and S2, Figures S1–S3, Tables S1–S3

Figure S4 Graphical abstract (‘Praatplaat’ in Dutch)
