# Supplementary material for: PENELOPE-CTRL: protocolised LDL-C lowering compared to real-world care in patients after myocardial infarction
Source: Neth Heart J. 2025 Jul 1;33(9):261–3. doi: 10.1007/s12471-025-01964-1 (PMC12364764; doi:10.1007/s12471-025-01964-1)
Supplement: Supplementary file 1 — Boxes S1 and S2, Figures S1–S3, Tables S1–S3 [file 12471_2025_1964_MOESM1_ESM.docx]

**Supplemental material**

**Box S1. PENELOPE-CTRL Trial Organisation**

**Steering Committee Members**

A.M.W. Alings, MD PhD FESC (chair)

F.M.A.C. Martens, MD PhD FESC

A.H. Liem, MD PhD

J.G.P. Tijssen, PhD FESC

A. Schut, MSc.

**Clinical Coordinating Center**

*Dutch Network for Cardiovascular Research, Utrecht, The Netherlands*

Operational Leadership: A. Schut

Project Manager: J. Kooistra

Data Managers: S. Hayen, A. de Vos and M. Atazadah

**CTRL Study Sites (number of patients included) and Investigators**

Isala Hospital, Meppel (122): P.A.M. Hoogslag, M. de Hoop

Haga Hospital, Den Haag (105): A.P. Haasdijk, E.C. Karijodikoro

Treant Zorggroep, Emmen (95): L. Kleijn, L. Schaafsma

NoordWest Ziekenhuisgroep, Alkmaar (92): D.J. Boswijk, S. van Lieshout

Haaglanden Medical Center, Den Haag (71): D.J. van der Heijden, I. Pardi

Canisius-Wilhelmina Hospital, Nijmegen (67): A.J.M. Oude Ophuis, I. Oving

Reinier de Graaf Gasthuis, Delft (58): J. Constandse, J. van Driel

Alrijne Hospital, Leiderdorp (53): T. J. Römer, A. van Dijk - van der Zanden

Deventer Hospital, Deventer (51): A. van der Sluis, W. Tousain

Diakonessenhuis, Utrecht (49): J. Houtgraaf, N. Plaizier

Máxima Medical Center, Veldhoven (24 ): S. Eijsbouts, I. Groenenberg

Elkerliek Hospital, Helmond (22): F.J. Prins, N. Nooijen - van de Kerkhof

Saxenburgh Medical Center, Hardenberg (18): J.T. Drost; E. Meijer

**
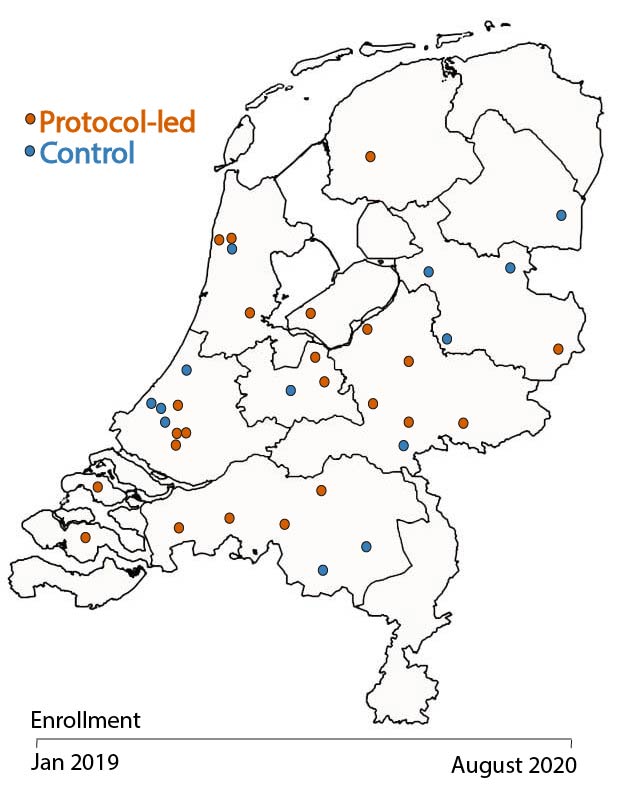
**

**Figure S1.** Geographical distribution of included sites in protocol group (PENELOPE) and control group (CTRL).

**Box S2. Detailed methods**

**Design**

The PENELOPE-CTRL was designed as a retrospective cohort study to serve as a real-world control cohort, with its enrolment-period matching the inclusion period of PENELOPE, i.e. January 1st, 2019 until August 31st, 2020. The PENELOPE study was an investigator-initiated, prospective, non-randomised trial, conducted within 23 non-academic centres across the Netherlands. In very high-risk patients presenting with (non-)ST-elevation myocardial infarction ((N)STEMI), the prevalence of patients with LDL-C ≤1.8 mmol/L was determined at baseline, after adding high-intensive statin (HIST) (step 1), after adding ezetimibe (step 2), and after adding PCSK9i (step 3). In PENELOPE, LDL-C was monitored at baseline and 4-6 weeks after every step, until LDL-C ≤1.8 mmol/L was reached. Additionally, in all patients that resigned consent, a single LDL-C measurement after one year was measured. The current study aligned the LDL-C target of ≤1.8 mmol/L with the PENELOPE study, that was based on the 2016 ESC guideline applicable at the beginning of the study, and still corresponds with current applicable Dutch cardiovascular guidelines. In the real-world control cohort, from here referred to as CTRL, we collected continuous LDL-C measurements in real-world controls from 3 months prior to and 18 months past MI. Participating sites in CTRL were also members of the WCN research network and had not participated in PENELOPE.

**Study populations**

Except for a maximum age cap on 70 years, inclusion criteria were identical to the PENELOPE trial, i.e. patients admitted for a type I (N)STEMI and a history of diabetes mellitus or ASCVD. In PENELOPE, patients >70 years of age were required to have a clinical frailty score (CFS) ≤3. As retrospective electronic medical records data do not allow for accurate determination of the CFS, all patients >70 years were excluded in CTRL to ensure comparability between both groups. Anticipating on a PENELOPE cohort of N=663 ≤70 years of age, a subcohort used for this analysis referred to as PENELOPE≤70, we aimed to include an at least same-sized CTRL real-world comparison cohort. Exclusion criteria were identical (pregnant or lactating women), with the exception that patients using PCSK9i or with known intolerance for PCSK9i at baseline could be included in the CTRL cohort, but were excluded for the primary outcome analysis. Life expectancy <1 year was omitted as this could not be determined reliably from the medical records retrospectively.

**Outcomes and outcome definitions**

Primary outcome focused on comparing the prevalence of patients achieving an LDL-C level of ≤1.8 mmol/L between the PENELOPE≤70 and CTRL cohorts within 6 months post-MI, corresponding to the full implementation period of the protocol-led strategy. The main secondary outcome was a comparison made closest to one year post-MI, within a timeframe of 6 to 18 months post-MI.

Other secondary outcomes included the prevalence of patients reaching LDL-C target of ≤1.8 mmol/L or ≤1.4 mmol/L (aligning with stricter European guidelines, although not being the original study goal) at any time during follow-up and while using oral LLT only. The latter analysis aimed to compare all LDL-C measurements following implementation of steps 1 (statin) and/or 2 (ezetimibe) in the protocol-led strategy against CTRL patients who were exclusively on oral LLT within the initial 6 months. Additionally, the analysis compared the median of lowest LDL-C levels at the specified timepoints, and the time in days after admission to reach the target for the first time among those who were not already at the target upon baseline assessment.

**Statistical methods**

At baseline, comparisons between patient characteristics, pre-hospitalisation LLT usage, and attainment of LDL-C target levels were conducted between the PENELOPE≤70 and CTRL cohorts. Baseline LDL-C in PENELOPE≤70 was measured during hospitalisation, whereas for CTRL, it represented the LDL-C measurement closest to hospitalisation, within a window from 3 months before admission to 28 days after discharge. To assess for potential imbalances between the cohorts, a Mann Whitney U-test and Fisher exact test were used for non-normal distributed continuous and dichotomous variables, respectively. A propensity score adjustment was planned in the event of clinically important imbalances.

For the primary and main secondary outcomes, as defined earlier, the analysis involved comparing the prevalences of patients achieving an LDL-C level of ≤1.8 mmol/L at two distinct timepoints: within 6 months post-MI and within a timeframe of 6 to 18 months, closest to one year post-MI. This analysis employed the lowest LDL-C measurement available for each patient within those timeframes. Prevalence comparisons utilised a Fisher exact test, with 95% confidence intervals calculated using the Wilson score interval formula.

In the one-year analysis, missing LLT usage data were imputed based on the last known medication. Additionally, a sensitivity analysis was performed, where missing LDL-C data in the 6-18 months period were imputed with the most recent prior LDL-C value carried forward, to account for potential selection bias.

For the secondary outcome of patients reaching LDL-C target at any time throughout follow-up, all available LDL-C measurements were used. In PENELOPE≤70, this included LDL-C measurements at baseline, during the use of the protocol and at approximately one year follow-up. For CTRL, this included all LDL-C measurements between baseline and 18 months post-MI.

Additionally, in the tailored analysis to a subgroup of patients receiving oral LLT only, LDL-C measurements from patients using PCSK9i were excluded. In case of changes in the LLT regimen in CTRL, we considered LDL-C measurements taken at least 28 days after these adjustments to accurately assess the impact of the new drug combination on LDL-C levels.

**
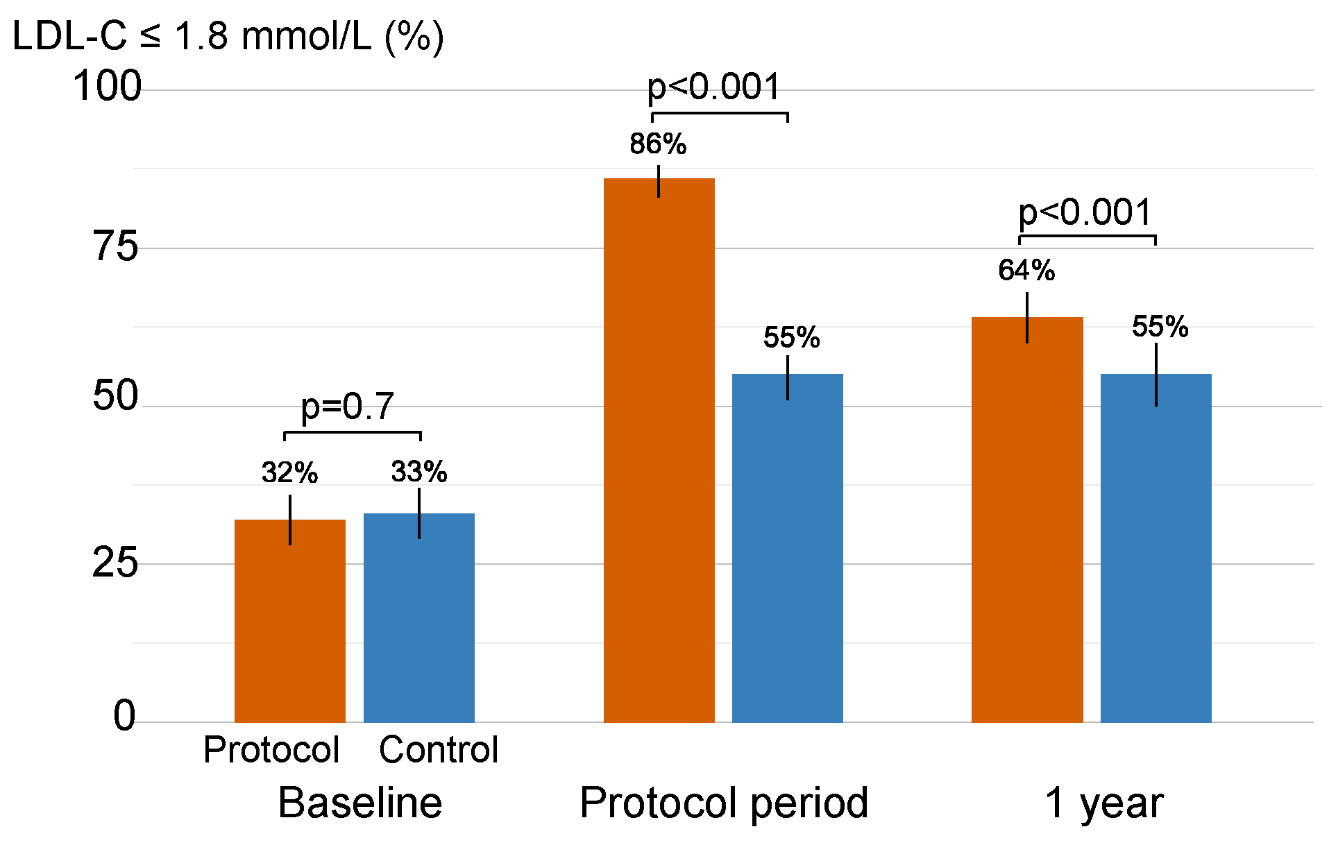
**

**Figure S2. LDL-C goal attainment with a short protocol-led LDL-C lowering strategy (PENELOPE) versus real-world controls (CTRL) in patients after hospitalisation for myocardial infarction, within the study period of 6 months and after approximately one year.**

**
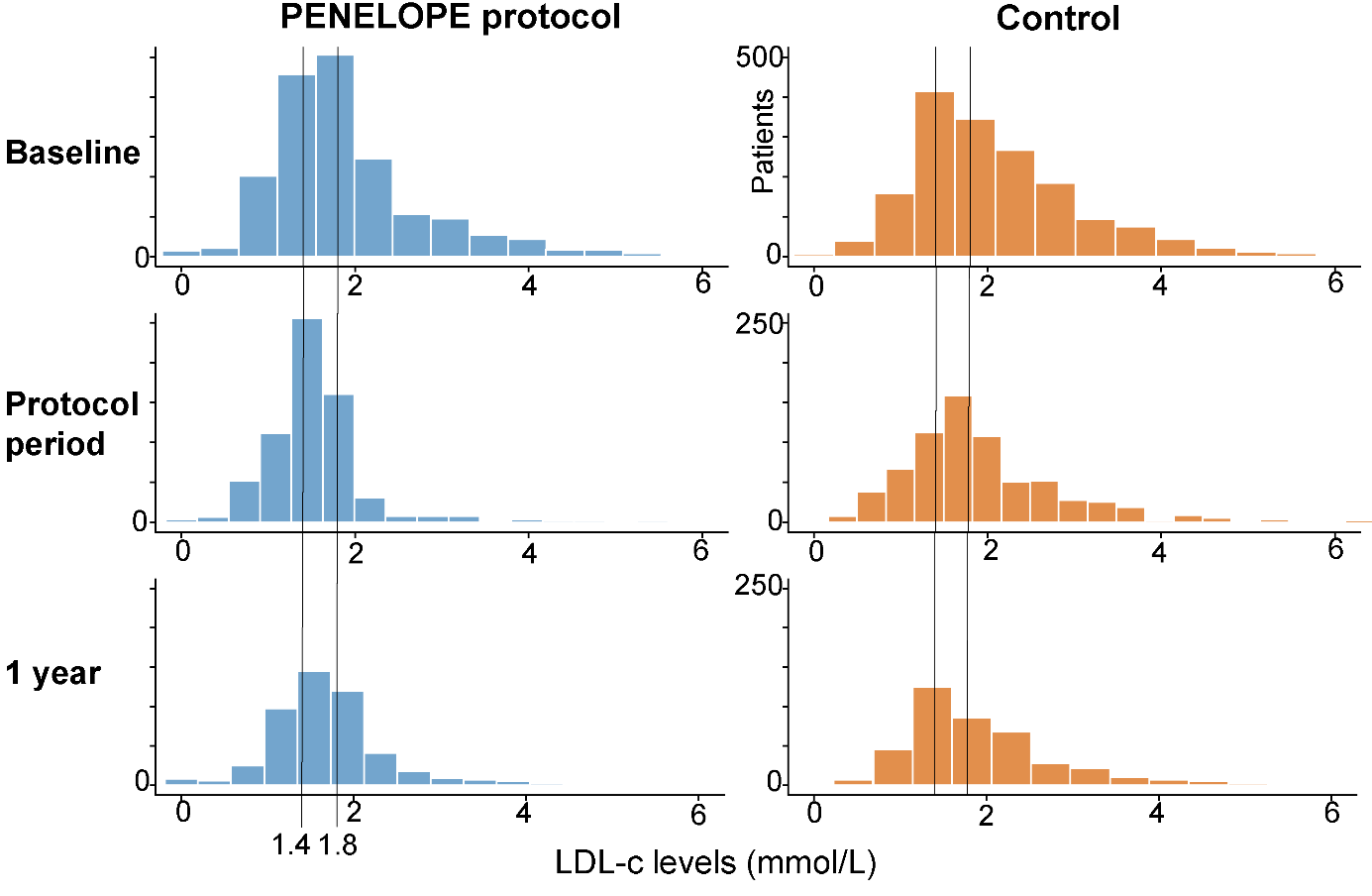
**

**Figure S3. Distribution of LDL-C levels with a short protocol-led LDL-C lowering strategy (PENELOPE) versus real-world controls (CTRL) in patients after myocardial infarction.** At baseline (hospitalisation for myocardial infarction), within the study period of 6 months and after approximately one year.

|  | | **PENELOPE-CTRL** | **PENELOPE**≤**70** | **p-value** |
| --- | --- | --- | --- | --- |
| **N** | | 827 | 663 |  |
| **Age, median (IQR)** | | 60 (54-64) | 62 (56-67) | <0.001 |
| **Women, n (%)** | | 198 (24) | 144 (22) | NS |
| **Index event, n (%)** | |  |  |  |
|  | Non-ST-elevation MI | 532 (64) | 438 (66) | NS |
|  | ST-elevation MI | 295 (36) | 225 (34) | NS |
| **Cardiovascular history, n (%)** | |  |  |  |
| Only DM type 2 | | 220 (27) | 198 (30) | NS |
| ASCVD | | 607 (73) | 465 (70) | NS |
|  | Coronary artery disease | 324/607 (53) | 269/465 (58) | NS |
|  | Cerebrovascular disease | 85/607 (14) | 74/465 (16) | NS |
|  | Aortic aneurysm or peripheral arterial disease | 71/607 (12) | 54/465 (12) | NS |
|  | With DMII | 164/607 (27) | 115/465 (25) | NS |
| **Risk factors, n (%)** | |  |  |  |
| Heart failure | | 37 (4) | 15 (2) | 0.02 |
| Hypertension | | 439 (53) | 374 (56) | NS |
| Smoking | | 348 (42) | 255 (38) | NS |
| Chronic kidney disease (eGFR<60) | | 43 (5) | 51 (8) | 0.05 |
| Family history of ASCVD | | 300 (36) | 257 (39) | NS |

**Table S1. Baseline characteristics.** Comparison between cohorts with Mann Whitney U-test for age (continuous non-normal distribution) and Fisher’s exact test for all other outcomes. Abbreviations: MI, myocardial infarction; DM, diabetes mellitus; ASCVD, atherosclerotic cardiovascular disease.

|  | | **PENELOPE-CTRL** | **PENELOPE**≤**70** | **p-value** |
| --- | --- | --- | --- | --- |
| **N** | | **827** | **663** |  |
| **Lipid lowering therapy, n (%)** | |  |  |  |
|  | PCKS9i | 13 (2) | NA |  |
|  | HIST + Ezetimibe | 24 (3) | 36 (5) | NS |
|  | HIST mono | 185 (22) | 146 (22) | NS |
|  | Non-HIST + ezetimibe | 29 (4) | 32 (5) | NS |
|  | Non-HIST mono | 314 (35) | 243 (37) | NS |
|  | Ezetimibe mono | 21 (3) | 18 (3) | NS |
|  | None | 241 (29) | 188 (28) | NS |
| **LDL-cholesterol levels** | |  |  |  |
| ≥1 LDL-C available in follow-up, n (%) | | 759 (92) | 663 (100) | <0.001 |
| Baseline LDL-C available, n/N | | 571/759 (76) | 642/663 (97) | <0.001 |
|  | median [IQR] | 2.3 [1.7-3.1] | 2.2 [1.6-3.0] | NS |
|  | ≤1.8 mmol/L | 181/571 (32) | 211/642 (33) | NS |
|  | ≤1.4 mmol/L | 85/571 (18) | 99/642 (15) | NS |

**Table S2. Lipid lowering management at baseline.** Abbreviations: PCKS9i, proprotein convertase subtilisin/kexin type 9 inhibitors; HIST; high-intensive statin; LDL-C; low-density lipoprotein cholesterol; IQR, interquartile range [25^th^-75^th^ percentile].

|  | | **PENELOPE-CTRL**  **N=827** | **PENELOPE**≤**70**  **N=663** | **p-value** |
| --- | --- | --- | --- | --- |
| **Lipid lowering therapy, n (%)** | |  |  |  |
|  | PCSKS9i (combi) | 55 (7) | 45 (7) | NS |
|  | HIST + Ezetimibe | 107 (13) | 151 (23) | <0.001 |
|  | HIST mono | 347 (42) | 264 (40) | NS |
|  | Non-HIST + ezetimibe | 61 (8) | 47 (7) | NS |
|  | Non-HIST mono | 198 (26) | 114 (17) | <0.001 |
|  | Ezetimibe mono | 30 (4) | 13 (2) | NS |
|  | None | 29 (4) | 29 (4) | NS |
| **LDL-cholesterol levels** | |  |  |  |
| One year LDL-c available, n | | 412 (50) | 490 (74) | <0.001 |
|  | median [IQR] | 1.9 (1.4-2.3) | 1.6 (1.3-2.0) | <0.001 |
|  | ≤1.8 mmol/L | 228/412 (55) | 314/490 (64) | 0.008 |
|  | ≤1.4 mmol/L | 124/412 (30) | 168/490 (34) | NS |
| Including imputation of last LDL-c, n | | 759 | 663 |  |
|  | median [IQR] | 1.7 (1.4-2.3) | 1.6 (1.3-1.9) | <0.001 |
|  | ≤1.8 mmol/L | 421/759 (55) | 445/663 (67) | <0.001 |
|  | ≤1.4 mmol/L | 240/759 (32) | 237/663 (36) | NS |

**Table S3. Lipid lowering management at approximately one year after myocardial infarction.** In 159 PENELOPE patients where lipid lowering treatment usage at one year was not available, it was imputed with the last lipid lowering treatment known. Abbreviations: PCKS9i, proprotein convertase subtilisin/kexin type 9 inhibitors; HIST; high-intensive statin; LDL-C; low-density lipoprotein cholesterol; IQR, interquartile range [25^th^-75^th^ percentile].
