# Supplementary material for: PENELOPE-CTRL: protocolised LDL-C lowering compared to real-world care in patients after myocardial infarction
Source: Neth Heart J. 2025 Jul 1;33(9):261–3. doi: 10.1007/s12471-025-01964-1 (PMC12364764; doi:10.1007/s12471-025-01964-1)
Supplement: Supplementary file 2 — Figure S4 Graphical abstract (‘Praatplaat’ in Dutch) [file 12471_2025_1964_MOESM2_ESM.pdf]

In de PENELOPE en de PENELOPE-CTRL studies is gekeken hoeveel patiënten met en zonder LDL-C protocol binnen 6 maanden tot 1 jaar de targetwaarde ≤ 1.8 mmol/L bereiken.

LDL-C management na hartinfarct: op één lijn

Protocol gestuurd LDL management na myocardinfarct

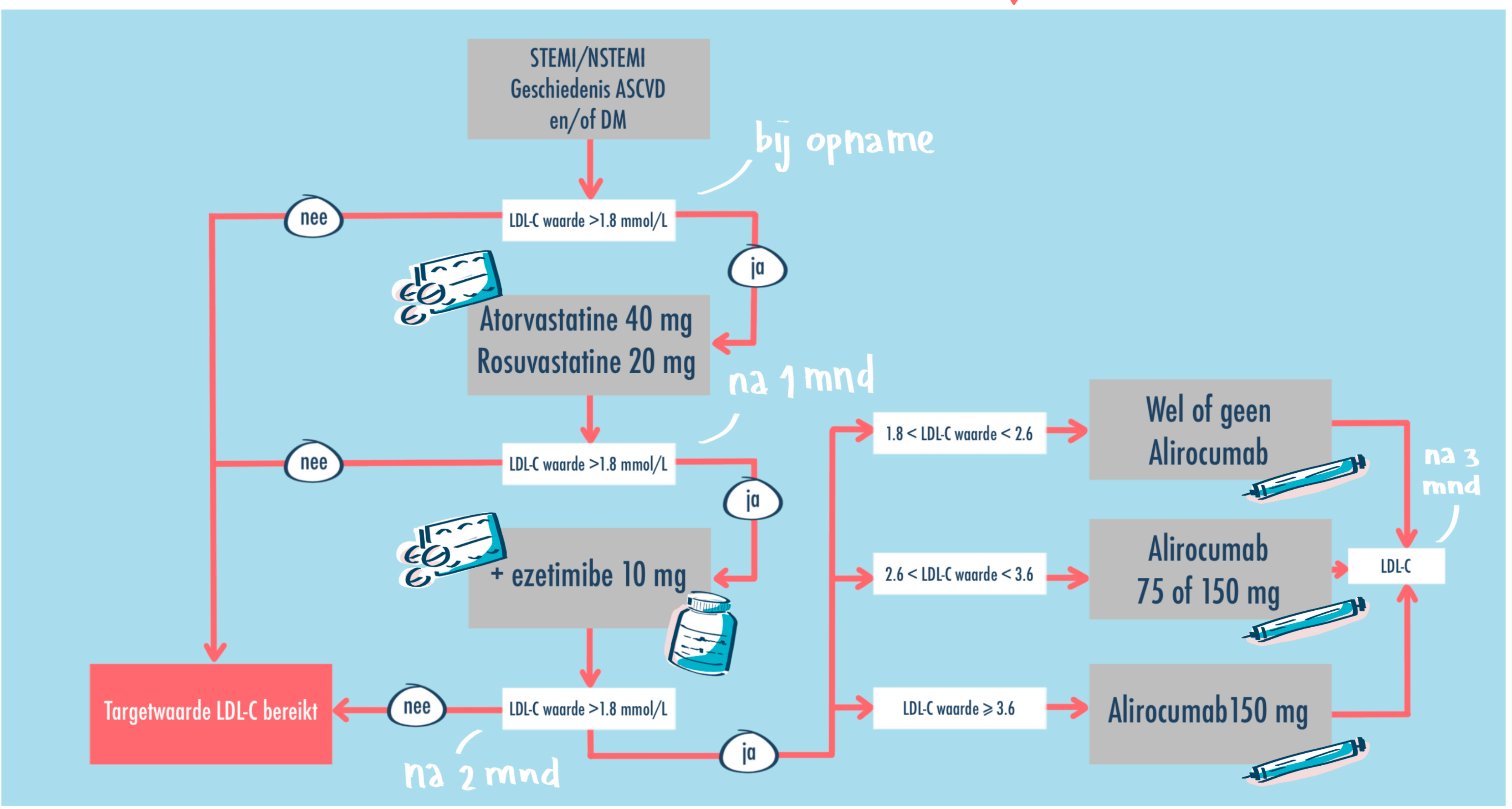

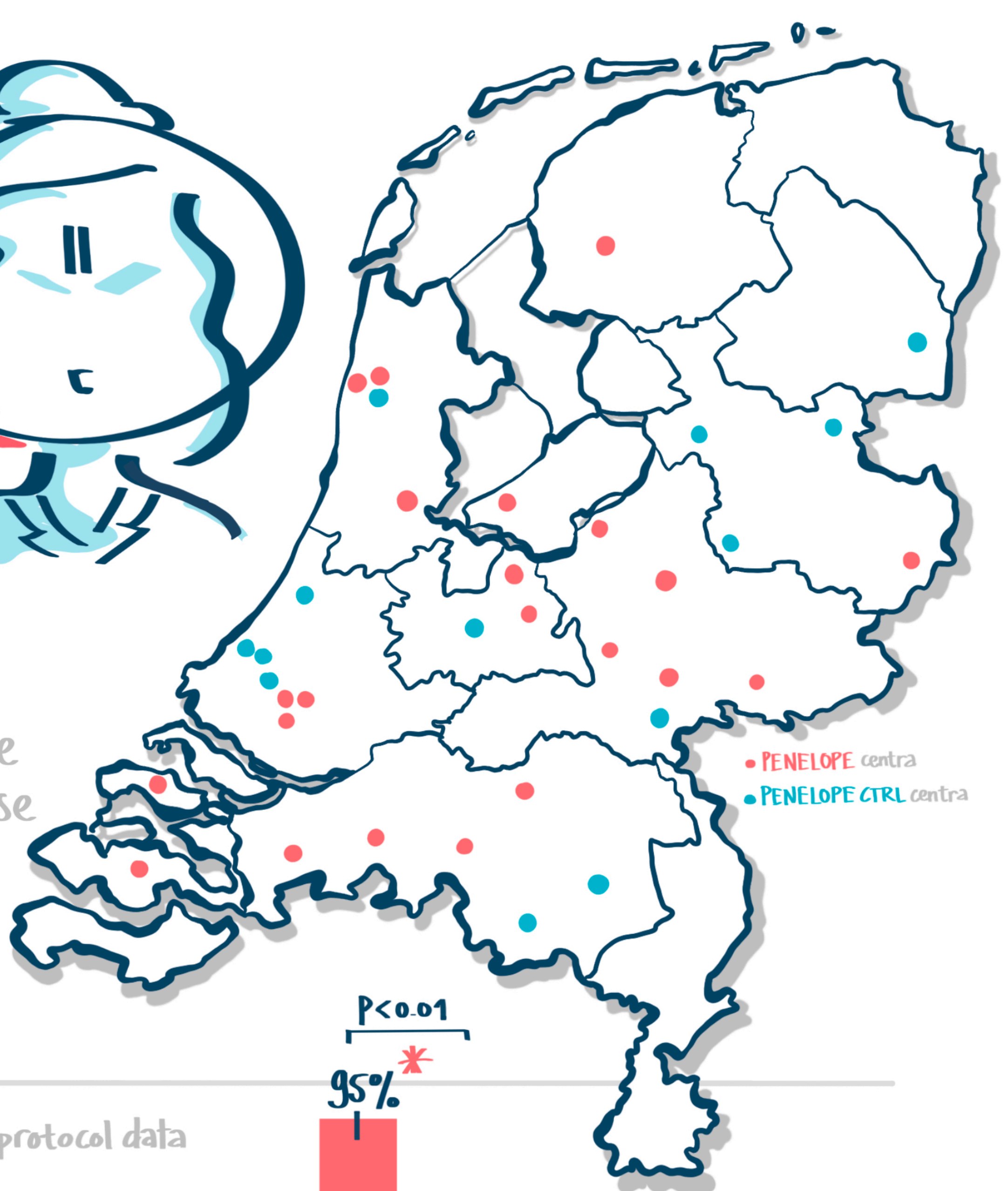

protocol gestuurd

Penelope
663 patiënten
♀ 22% ♂ 78%

normale dagelijkse zorg

Penelope-CTRL
827 patiënten
♀ 24% ♂ 76%

Beide studies zijn in Nederland uitgevoerd (periode januari 2019 - augustus 2021)

Age 18 - 70 jaar oud

Myocard infarct

Voorgeschiedenis diabetes mellitus of hart- en vaatziekten

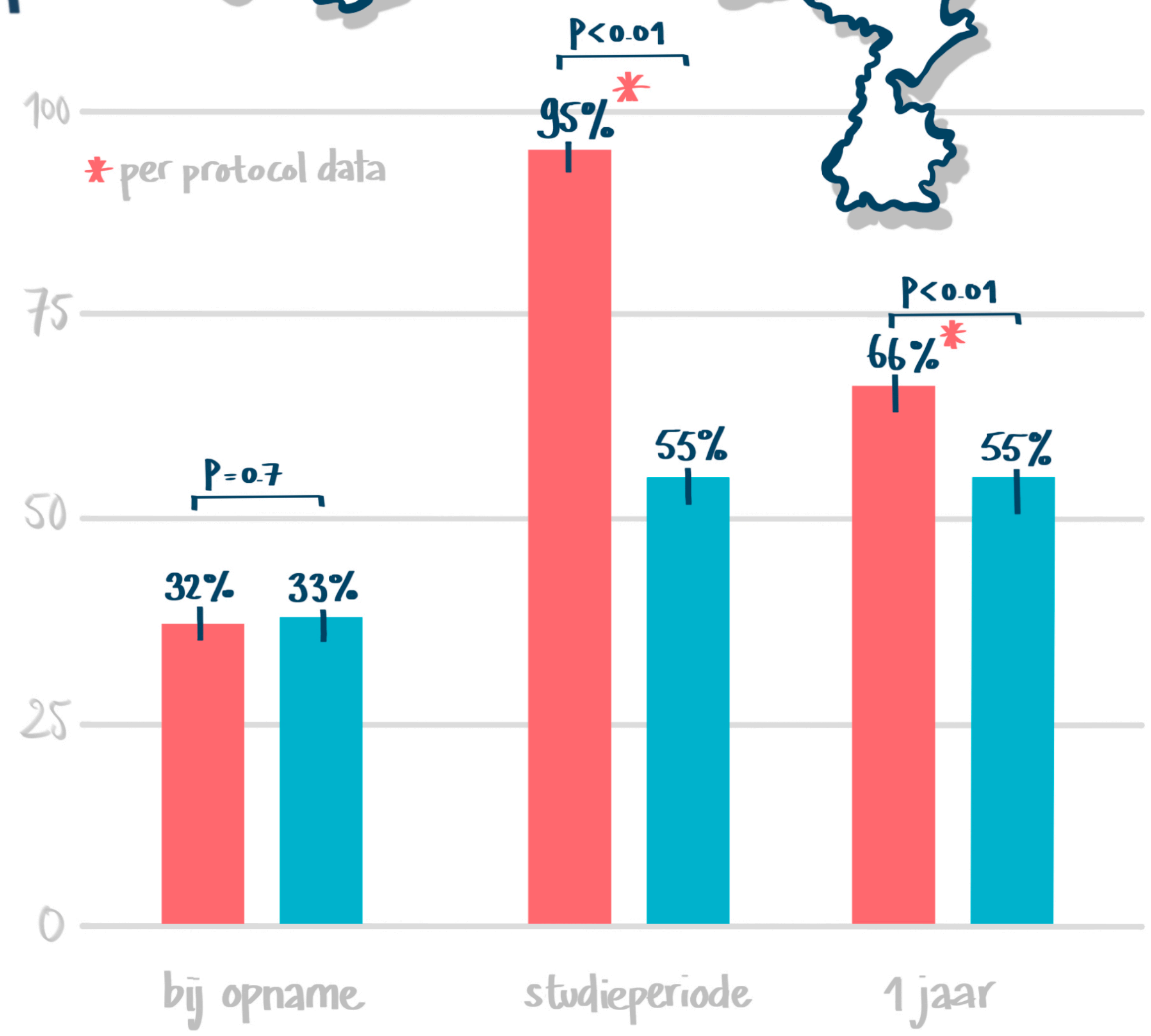

Percentage patiënten PENELOPE en PENELOPE CTRL op targetwaarde (1.8 mmol/L)

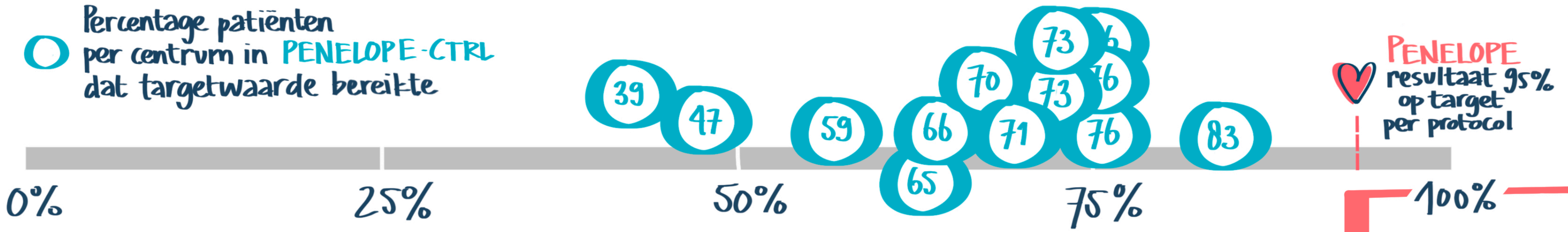

95% van de patiënten binnen 6 maanden op target door PENELOPE interventie.

Het PENELOPE effect neemt af in de dagelijkse zorg na een jaar, al blijft het gemiddelde LDL-C duidelijk lager.

Succes in de behandeling werd met name bereikt met orale medicatie (statines en ezetimibe), 4% van de patiënten gebruikte PCSK9i.

Dat PENELOPE protocol lijkt wel te werken. Of niet?

Paper PENELOPE: Effects of a stepwise, structured LDL-C lowering strategy in patients post-acute coronary syndrome. Aaram Omar Khader, Tinka van Trier, Sander van der Brug, An-ho Liem, Bjorn Groenemeijer, Astrid Schut, Harald Jorstad, Fabrice Martens, Marco Alings. Netherlands Heart Journal 2024
Paper PENELOPE 1 yr FU: PENELOPE one year follow-up: legacy effect of a short protocol-led LDL-C lowering strategy in patients after myocardial infarction. Sander van der Brug, Tinka van Trier, Aaram Omar Khader, An-ho Liem, Astrid Schut, Fabrice Martens, Marco Alings. Target journal: Netherlands Heart Journal
Paper PENELOPE-CTRL: PENELOPE-CTRL: LDL-C lowering in short protocol-led care compared to routine care in patients after myocardial infarction. Tinka van Trier, Aaram Omar Khader, Sander van der Brug, An-Ho Liem, Astrid Schut, Jan Tijssen, Fabrice Martens, Marco Alings.
Target journal: Netherlands Heart Journal

Clinical Operations WCN was de sponsor van PENELOPE en PENELOPE-CTRL en verantwoordelijk voor het trial design, site management en uitvoering van data analyses van beide studies. Dit onderzoek is ondersteund door een subsidie van Sanofi-Aventis Netherlands B.V.
